# Supplementary figures and images for: Supernumerary teeth observed in a live True’s beaked whale in the Bay of Biscay
Source: PeerJ. 2019 Oct 14;7:e7809. doi: 10.7717/peerj.7809 (PMC6796962; doi:10.7717/peerj.7809)

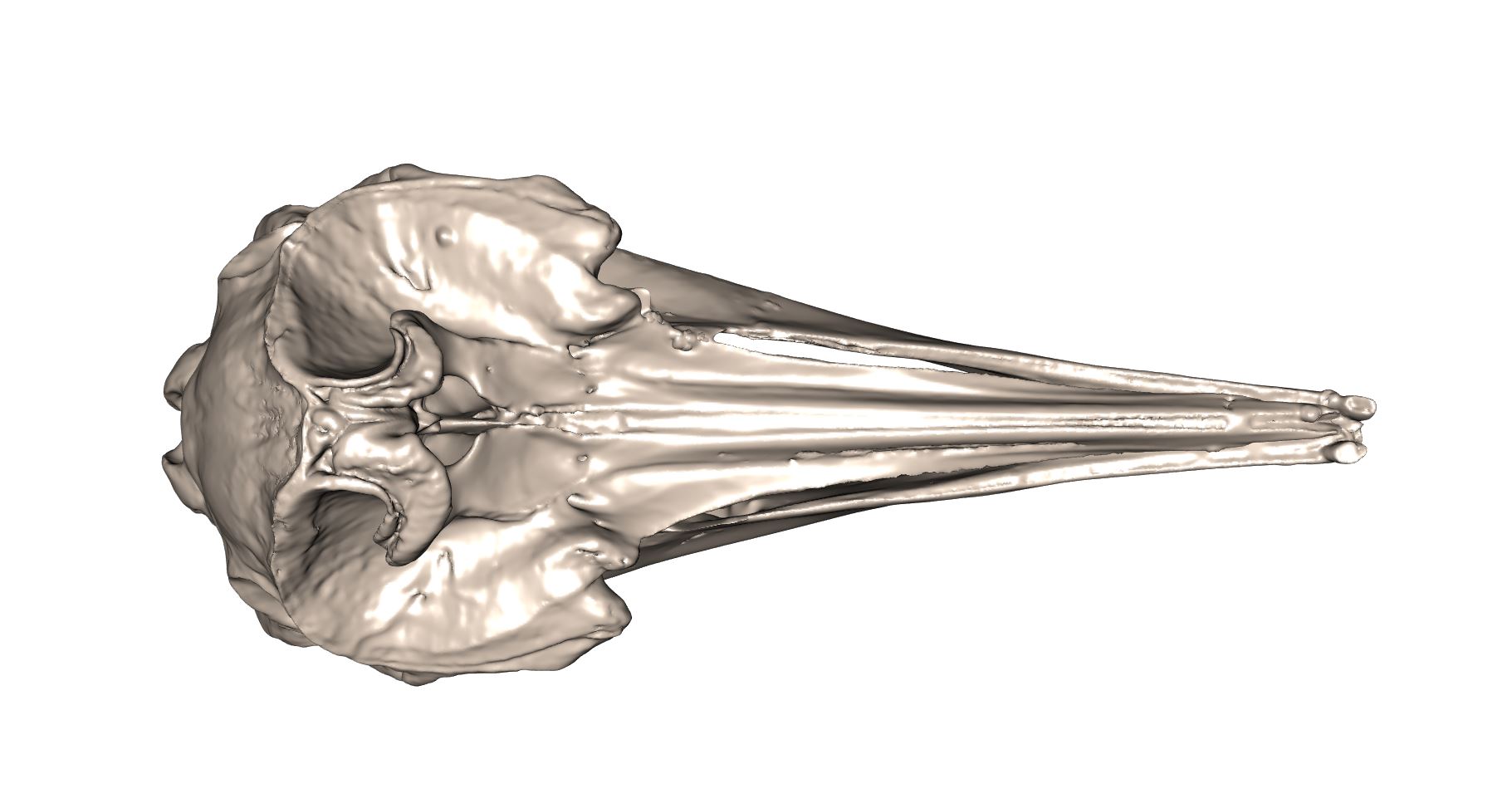

Supplement: Supplemental Information 1 — Captured in 3D using a Creaform Go!SCAN 50 laser scanner and VXElements software. Scans were cleaned, prepared, and exported to .ply in Geomagic Wrap software at a resolution of 0.8 mm and then rendered in MeshLab. The .ply file is available as supplementary material 2. [file peerj-07-7809-s001.jpg]
